# Missed Opportunity: The Unseen Driver for Low Coronavirus Disease 2019 Vaccination Rates in Underserved Patients

**DOI:** 10.1093/ofid/ofac326

**Published:** 2022-07-22

**Authors:** Tania Campagnoli, Geetika Mohan, Nigist Taddese, Yaveen Santhiraj, Natasa Margeta, Saad Alvi, Umair Jabbar, Aniesh Bobba, Jihad Alharash, Michael J Hoffman

**Affiliations:** Department of Medicine, Division of Hospital Medicine, John H. Stroger Jr., Hospital of Cook County, Chicago, Illinois, USA; Department of Medicine, Division of Hospital Medicine, John H. Stroger Jr., Hospital of Cook County, Chicago, Illinois, USA; Department of Medicine, Division of Hospital Medicine, John H. Stroger Jr., Hospital of Cook County, Chicago, Illinois, USA; Department of Medicine, Division of Hospital Medicine, John H. Stroger Jr., Hospital of Cook County, Chicago, Illinois, USA; Department of Medicine, Division of Hospital Medicine, John H. Stroger Jr., Hospital of Cook County, Chicago, Illinois, USA; Department of Medicine, Division of Hospital Medicine, John H. Stroger Jr., Hospital of Cook County, Chicago, Illinois, USA; Department of Medicine, Division of Hospital Medicine, John H. Stroger Jr., Hospital of Cook County, Chicago, Illinois, USA; Department of Medicine, Division of Hospital Medicine, John H. Stroger Jr., Hospital of Cook County, Chicago, Illinois, USA; Department of Medicine, Division of Hospital Medicine, John H. Stroger Jr., Hospital of Cook County, Chicago, Illinois, USA; Department of Medicine, Division of Hospital Medicine, John H. Stroger Jr., Hospital of Cook County, Chicago, Illinois, USA

**Keywords:** African American, COVID-19 vaccination, Latino, vaccine hesitancy

## Abstract

**Background:**

Vaccines against severe acute respiratory syndrome coronavirus 2 (SARS-CoV-2) virus have been available since December 2020. Vaccination rates among hospitalized patients at our institution remained low at approximately 40%, thus we sought to understand the drivers of vaccine hesitancy in our patient population.

**Methods:**

All unvaccinated adult patients admitted to our hospital were asked to participate in a survey to assess coronavirus disease 2019 (COVID-19) vaccine hesitancy. Updated vaccination status was collected at the end of the study.

**Results:**

Ninety-seven patients agreed to participate, 34% of which were SARS-CoV-2 positive based on results from polymerase chain reaction tests. Of the 64 participants eligible to receive the vaccine, 57.8% were agreeable but only 27% received the vaccine before discharge.

**Conclusion:**

Many patients are willing to receive the vaccine, and hospitalization provides a unique opportunity to interact with patients who have been otherwise unaware, unable, or unwilling to pursue vaccination outside of the hospital.

Since the first reported case of coronavirus disease 2019 (COVID-19), the resulting global pandemic has led to over 77 million infections and close to 1 million deaths in the United States [1]. Vaccines against severe acute respiratory syndrome coronavirus 2 (SARS-CoV-2) have been available since December 2020. The vaccination rate in the United States is approximately 65% [1, 2]. However, our local experience in our urban safety-net hospital shows a vaccination rate through February 2022 of approximately 40%.

Unvaccinated adults are 3 times more likely to test positive for COVID-19 and are 16 times more likely to get hospitalized compared with those fully vaccinated [3]. With the emergence of SARS-CoV-2 variants rechallenging our healthcare system, it is critical that we improve vaccination rates across our communities to reduce transmission, severity of illness, and to mitigate hospitalizations.

Our hospital has offered inpatient vaccination since April 2021; however, the vaccination rate is low. Given the substantial proportion of unvaccinated patients admitted to our hospital and the low rate of vaccination, we sought to understand the drivers for vaccine hesitancy and assess for opportunities to improve COVID-19 vaccination rates among our patients.

## METHODS

### Study Participants

Adult patients (>18 years of age) admitted to the medical/surgical wards from January 10, 2022 to February 6, 2022 were screened for their COVID-19 vaccination status. All unvaccinated patients were offered the opportunity to participate in the survey. Patients with encephalopathy, dementia, or those currently incarcerated were excluded. The survey was conducted by a physician, either in person or by phone.

### Patient Consent

Oral informed consent was obtained for all participants. The design of the study has been approved by the local ethical committee.

### Measurements

We developed a survey to understand drivers of vaccine hesitancy in study participants. We collected demographic, economic, and social data as seen in Table 1. Race and ethnicity were self-identified. We also inquired about COVID-19 infection history and rationale behind their unvaccinated status, and we assessed patients’ willingness to obtain a COVID-19 vaccination and discussed entities that patients trusted to provide them with accurate information about the COVID-19 vaccines.

**Table 1.** Sociodemographic Characteristics, Access to Care, and Trust in Healthcare

Characteristics	Total Sample, n (%)	Willing to Get Vaccination	
		Yes, n (%)	No, n (%)
Gender			
Male	64 (66)	41 (64)	23 (36)
Female	33 (34)	22 (66)	11 (34)
Age			
18–24	7 (7)	4 (57)	3 (43)
25–34	15 (16)	7 (46)	8 (54)
35–49	20 (21)	14 (70)	6 (30)
50–64	45 (46)	33 (73)	12 (27)
>65	10 (10)	5 (50)	5 (50)
Race/Ethnicity			
Black	56 (58)	35 (62)	21 (38)
Hispanic	26 (27)	21 (81)	5 (19)
White	14 (14)	6 (43)	8 (57)
Asian	1 (1)	0 (0)	1 (100)
Homeless			
Yes	92 (95)	60 (65)	32 (35)
No	5 (5)	3 (60)	2 (40)
Household Size			
1	18 (19)	12 (67)	6 (33)
2–4	59 (64)	36 (61)	23 (39)
>5	16 (17)	13 (81)	3 (9)
Level of Education			
<8th	8 (8)	7 (87.5)	1 (12.5)
8–12th	58 (60)	37 (64)	21 (36)
College/University	31 (32)	19 (61)	12 (39)
Employed			
Yes	40 (41)	25 (62.5)	15 (37.5)
No	57 (59)	38 (67)	19 (33)
Occupational Exposure			
Yes	30 (31)	22 (73)	8 (27)
No	66 (69)	40 (61)	26 (39)
Past COVID-19 Infection			
Yes	20 (21)	10 (50)	10 (50)
No	77 (79)	53 (69)	24 (31)
Know Someone Who Suffered Severe COVID-19			
Yes	43 (46)	26 (60)	17 (40)
No	51 (54)	37 (72.5)	14 (27.5)
Seen a Doctor in the Last 6 Months			
Yes	47 (49)	26 (55)	21 (45)
No	50 (51)	37 (74)	13 (26)
Talked to a Doctor About Vaccination			
Yes	34 (38)	20 (59)	14 (41)
No	55 (62)	38 (69)	17 (31)
Trust Doctors to Deliver Information About COVID-19			
Yes	45 (53)	34 (75)	11 (25)
No	40 (47)	23 (57.5)	17 (42.5)
Healthcare Insurance			
Yes	67 (69)	39 (58)	28 (42)
No	30 (31)	24 (80)	6 (20)

Abbreviations: COVID, coronavirus disease 2019.

NOTE: Of the 97 patients that participated in the survey, 63 patients were willing to get the vaccine versus 34 patients unwilling. Patient demographics and pertinent answers are documented in this table. Percentage in willingness or unwillingness of vaccination is according to characteristic. Not everyone completed the full survey.

For patients interested in being vaccinated, the primary team was contacted and made aware of this interest. Vaccine administration data was then collected at the end of the study period. For patients with active COVID-19 infection, vaccination was recommended once they recovered from acute infection.

### Data Analysis

IBM SPSS statistics was used to analyze the data. Descriptive statistics, frequencies, and percentages were calculated to describe the cohort, and data were then separated according to vaccine willingness for comparison of the groups.

## RESULTS

### Participant Characteristics

Of the 124 patients approached, 97 (78.2%) agreed to participate. Of those surveyed, 34% tested positive for COVID-19 via polymerase chain reaction (PCR) testing. As seen in Table 1, the majority (66%) were male. The largest proportion of patients surveyed identified as Black at 58%, followed by Hispanic at 28% and White at 14%. Consistent with our patient population, 5% were homeless, and 20% were not US citizens or permanent residents. Some form of healthcare insurance was available to 70% of patients. The majority were unemployed (60%). Only approximately 50% reported seeing a doctor in the prior 6 months.

### Acceptance of COVID-19 Vaccine

Sixty-four study participants had a negative SARS-CoV-2 PCR on admission, making them eligible to get vaccinated during the same hospitalization. Of these, 57.8% were agreeable to receive the COVID-19 vaccine; however, only 27% received it before hospital discharge.

Comparing participants willing to those unwilling to be vaccinated (Table 1), we noted that those between the ages of 50 and 60 years were most willing to receive the vaccine (73%). There was a difference among race/ethnicity as well in that 80% of Hispanic participants agreed to be vaccinated versus 62% of Black participants and 40% of White participants. Patients with a household size of more than 5 people were more likely to agree to get vaccinated versus rejecting the vaccine.

Of the 63 participants willing to get vaccinated, 58.7% had not seen a doctor in the past 6 months and 65.6% had not spoken to a healthcare worker about the vaccine. Of the 45 participants that reported trust in healthcare workers, 75% agreed to get vaccinated.

Trusted sources included physician (53%), news (31%), and family and friends (28%). Eight percent trusted no one and only 19% trusted the internet.

### Drivers for Low COVID-19 Vaccination Rates

Of those stating their willingness to receive the vaccine, the main driver of being unvaccinated appears to be missed opportunity. This takes several forms in that the majority either did not have access to a doctor or did not see a doctor recently, and some had a poor understanding of whether the vaccine was safe for them given their other medical conditions. Others felt they did not have easy access to the vaccine and thus wanted to take the opportunity to be vaccinated while in the hospital. Of those still not willing to receive the vaccine, the main reasons included fear of short- and long-term side effects, not enough research on the COVID-19 vaccines, the belief that it is not effective, and government mistrust.

## DISCUSSION

Eighty percent of our unvaccinated patients surveyed were Black and Hispanic. Our finding of greater vaccine hesitancy among these minority groups has been shown in previous studies [4, 5] and are in accordance with the report of the Cook County Department of Public Health, which shows that vaccination rates in the entirety of Cook County, Illinois are lower in Black (64%) and Hispanic (65%) populations compared with Whites (79%) [6]. These differences could be related to distrust of the medical establishment and government, especially in the Black population [7]. It is interesting to note, however, that 81% of Hispanic and 62% of Black participants agreed to get vaccinated before discharge home, leading us to believe this could be more of an access issue than absolute vaccination refusal—a truly missed opportunity.

The age groups most willing to receive the vaccine were 50–60 years (73%) followed by 35–49 years (70%). Vaccine uptake may vary among different age groups. For instance, Luyten et al [8] found that adults aged 50–59 had more vaccine confidence than those aged 20–29. The high rate of willingness in our patients in this age group may be because they have more barriers to access of the vaccine related to comorbidities, transportation, technology, and employment-related issues. This population will benefit from vaccination at any time of healthcare contact.

Participants reported that a reliable source discussing the research and benefits of the vaccine would change their mind about receiving the vaccine. This is illustrated by 53% of all participants and indicated that they trust physicians to give them reliable information about the vaccine. This finding is encouraging and previously documented [9–11]. Another missed opportunity is seen here in that those surveyed are currently under the care of inpatient physician teams, but COVID-19 vaccination had not been discussed with these patients. Inpatient teams are managing multiple complex medical problems and therefore may not prioritize discussing COVID-19 vaccination status with their patients. Our study highlights that missed opportunities for healthcare providers recommending and administering vaccines to patients are still common and that physician-prompted discussions regarding the COVID-19 vaccine, in an inpatient setting, can be effective in increasing vaccination rates.

Half of our study subjects do not see a doctor regularly. Establishing them with a primary care physician would give them the opportunity to discuss COVID-19 vaccination and address their hesitancy with a reliable source, thereby increasing the likelihood of accepting the vaccine. It is unfortunate that the shift from in-person clinic visits to telehealth visits during the pandemic has likely led to decreased opportunities to speak with healthcare providers and receive the vaccine in a convenient manner.

Despite our efforts, only 27% of patients agreeable to get the vaccine received the vaccine during their hospitalization. There are likely several reasons for this. The physician team is likely more focused on the reason for hospitalization and did not prioritize COVID-19 vaccination or believed that it may be contraindicated during this hospitalization. In addition, the logistics of receiving the COVID-19 vaccine while hospitalized requires significant forethought and planning to reduce vaccine waste given the limited shelf life of the vaccine. Healthcare systems need to work on logistics to make the vaccine more accessible to patients so as not to miss more opportunities to vaccinate those who are willing.

## CONCLUSIONS

It is long known that the physician-patient relationship is critical. Our study confirms that our patients do, in fact, trust their healthcare providers. It is unfortunate that many of them have not had access to a physician, and thus this important bond has not been able to be leveraged to increase vaccination rates among some of the most vulnerable. Physicians of all specialties should have the knowledge and ability to talk to patients about the COVID-19 vaccine at all points of contact to increase vaccine uptake. Although COVID-19 vaccination of eligible inpatients has logistical and clinical challenges, it provides a unique opportunity to interact with patients who have not had access to a physician and have been otherwise unaware, unable, or unwilling to pursue vaccination outside of the hospital. We must seize this so as not to miss another opportunity.
